# Development of a highly controlled system for large-area, directional printing of quasi-1D nanomaterials

**DOI:** 10.1038/s41378-021-00314-6

**Published:** 2021-10-19

**Authors:** Adamos Christou, Fengyuan Liu, Ravinder Dahiya

**Affiliations:** Bendable Electronics and Sensing Technologies (BEST) Group, James Watt School of Engineering, University of Glasgow, Glasgow, G12 8QQ UK

**Keywords:** Electrical and electronic engineering, Nanowires

## Abstract

Printing is a promising method for the large-scale, high-throughput, and low-cost fabrication of electronics. Specifically, the contact printing approach shows great potential for realizing high-performance electronics with aligned quasi-1D materials. Despite being known for more than a decade, reports on a precisely controlled system to carry out contact printing are rare and printed nanowires (NWs) suffer from issues such as location-to-location and batch-to-batch variations. To address this problem, we present here a novel design for a tailor-made contact printing system with highly accurate control of printing parameters (applied force: 0–6 N ± 0.3%, sliding velocity: 0–200 mm/s, sliding distance: 0–100 mm) to enable the uniform printing of nanowires (NWs) aligned along 93% of the large printed area (1 cm^2^). The system employs self-leveling platforms to achieve optimal alignment between substrates, whereas the fully automated process minimizes human-induced variation. The printing dynamics of the developed system are explored on both rigid and flexible substrates. The uniformity in printing is carefully examined by a series of scanning electron microscopy (SEM) images and by fabricating a 5 × 5 array of NW-based photodetectors. This work will pave the way for the future realization of highly uniform, large-area electronics based on printed NWs.

## Introduction

The rapidly increasing demand for realizing electronics in flexible and deformable form factors and over large areas is challenging to meet with conventional micro/nanofabrication manufacturing techniques^1^. This is due to the inherent limitations of conventional methods, which make them more suitable for realizing electronics on planar substrates. Fabrication methods that adapt to the needs of the new generation of high-performance flexible electronics while striving to achieve cost-effective high-throughput production are needed. To this end, printed electronic technologies have emerged as a promising alternative. They offer low operational complexity, reduced chemical waste, a low processing temperature, and excellent compatibility with many unconventional substrates^1–5^.

In recent years, various printing techniques have emerged for fabricating flexible electronics based on both organic and inorganic materials^1,4,6–8^. Among the various developed printing methods, contact printing holds great promise for large-area, high-performance flexible electronics based on quasi-one-dimensional (quasi-1D) materials. With contact printing, it is possible to print various types of quasi-1D materials with pre-defined alignment onto target substrates at room temperature. The dry nature of the process along with its low-temperature capability allows for a broad range of materials, including flexible materials, which can be used as receiver substrates. The process also offers high positional accuracy, which can be further improved by surface functionalization and pre-patterning^9–12^. Furthermore, shear force-induced directional printing can also be realized in a roll-to-roll (R2R) manner, which is favorable for high-throughput manufacturing^13–17^.

Although the concept of contact printing and its variants, such as differential roll printing, have been reported in the past^10,11,18–32^, there is limited information available on the equipment that is used to carry out the process. Often, manual approaches have been employed, such as pressing by hand or using weights^11,18^, whereas in other cases, equipment meant for other uses was repurposed^22^. This study presents a well-controlled system for implementing contact printing, with a detailed report on the development of a custom-built system that implements the contact printing process. Specifically, the system allows for precise and independent control over the printing process parameters, thus enabling systematic testing and optimization of the process. The developed system is fully automated, thus minimizing human-induced variation in the printing process. The advancements described in this study include the following: (a) a novel and more reliable mechanism for substrate alignment and (b) improved automated processing. The former leads to increased printing uniformity over large areas, whereas the latter limits the risk of manual error and facilitates systematic printing and testing. A detailed study was carried out to illustrate the uniformity of the printed nanowires (NWs) via a series of scanning electron microscopy (SEM) characterizations across the entire area covered by the donor substrate. To further demonstrate the printing uniformity and its significance for large-area manufacturing, an array of ultraviolet (UV) photodetectors was fabricated using printed NW layers. By developing and using a highly controlled system, such as the one presented here, the contact printing method process can be better examined and understood and could be optimized to meet the emerging industry requirements for large-area printed electronics.

## Results

### Concept

**Fig. 1:**
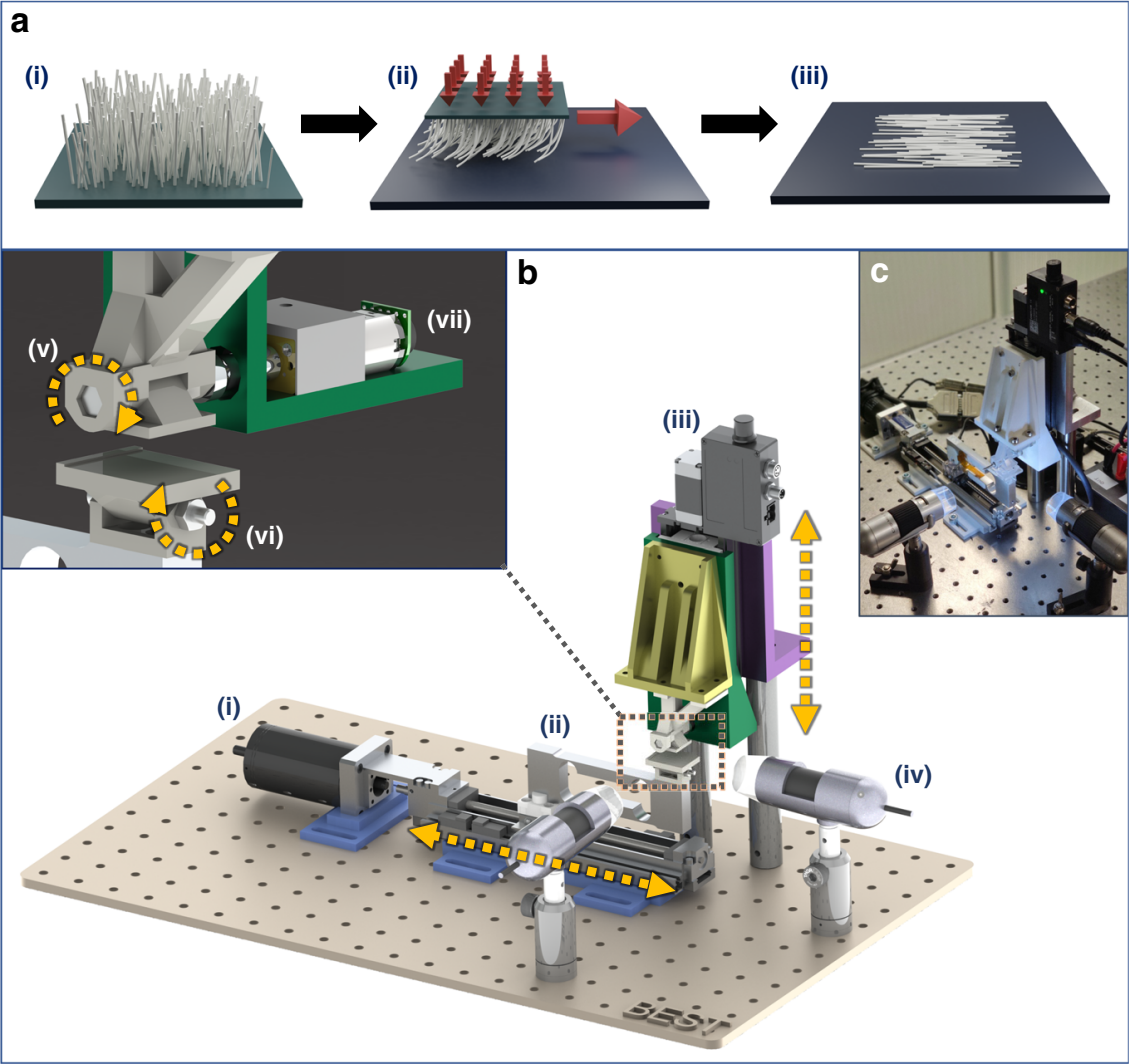
Design of custom-made system for highly controlled contact printing of nanowires. **a** Steps of the contact printing process: i donor substrate with vertically grown nanowires; ii sliding of the donor substrate across the receiver substrate, while also applying pressure; iii printed nanowires on the receiver substrate aligned along the direction of sliding. **b** 3D model of the designed contact printing system: i horizontal actuator; ii load cell; iii vertical actuator; iv microscopes for alignment monitoring; v self-aligning donor substrate platform (primary axis); vi self-aligning receiver substrate platform (secondary axis); and vii mechanism for fixing the tilt of the platform. **c** Image of the assembled system

The contact printing process relies on direct contact between the donor and receiver substrates, whereas the normal and shear forces between two substrates enable the transfer of functional materials from one substrate to the other substrate. Figure 1a illustrates the steps involved in the contact printing process. Initially, a donor substrate, usually consisting of vertically grown NWs on a flat rigid substrate, is brought into contact with a receiver substrate. Pressure is applied between the two and, subsequently, the donor substrate slides across the receiver while maintaining the applied pressure. Finally, the donor substrate is lifted from the receiver. Through this process, the initially vertical NWs on the donor substrate are detached and transferred flat on the surface of the receiver substrate aligned along the sliding direction. Controlled alignment can benefit the device fabrication process and achievable performance, as it reduces NW overlaps and enhances the uniformity of the electronic layer. The contact printing technique is a simple and effective technique to achieve transfer and alignment in a single step.

### System design

The developed contact printing system depicted in Fig. 1b, c comprises two main actuating platforms: the vertical platform, which applies the lateral force on the substrates (Fig. 1b-iii), and the horizontal platform, which controls the shear force (Fig. 1b-ii). A load cell is attached to the moving horizontal platform and is used to monitor the applied force during printing (Fig. 1b-ii).

**Fig. 2:**
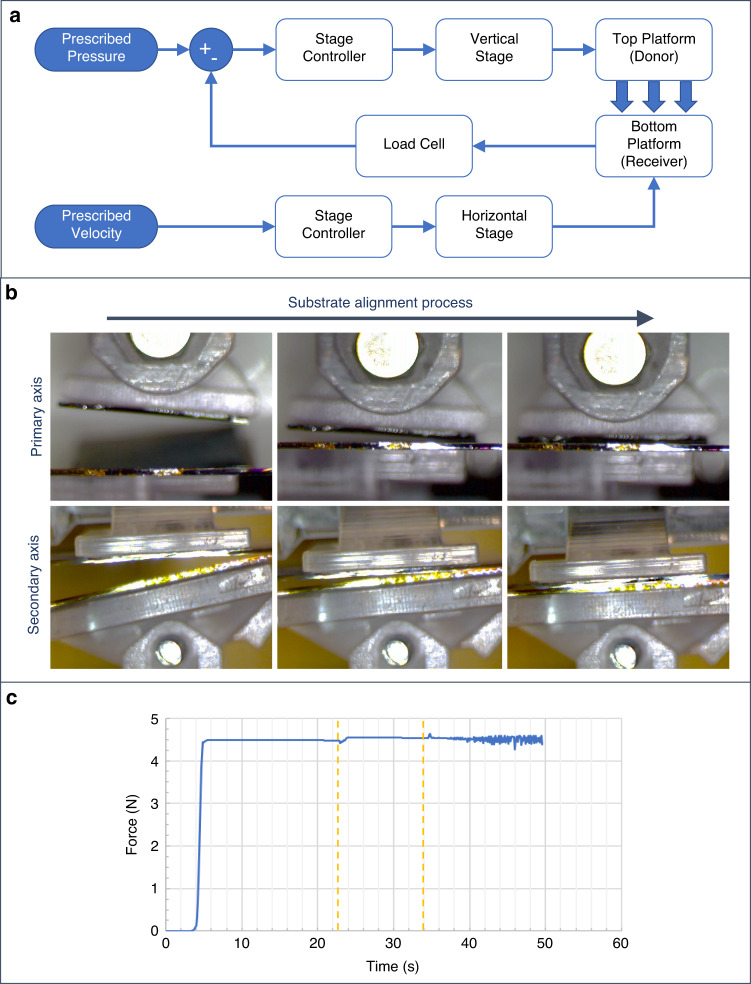
Operation of highly controlled contact printing system. **a** Block diagram of closed-loop control implemented by the software. **b** Self-aligning action of the substrate platforms when force is applied. **c** Plot of applied force during printing showing minimal fluctuation. The highlighted region corresponds to the optional fixing of the tilting platform

The donor and receiver substrates are placed at the interface of the two moving platforms. To make use of the entire area of the donor substrate and to achieve uniform printing, it is critical that the donor and receiver substrates tightly contact each other throughout the printing process. In the case of flat rigid substrates, planar alignment is required, which is challenging to achieve, particularly when the process is carried out manually. It is also challenging to maintain the alignment during the sliding step due to the high shear forces involved. If substrate alignment is disturbed during printing, the uniformity of the electronic layer from the printed nanostructures is affected, as parts of the donor are no longer in contact and thus not utilized. In addition, the uncontrolled change in the contact area can alter the applied pressure and further impact the printing performance (see the following section). To overcome these challenges, the presented custom-built contact printing system uses a new mechanism that allows the two substrate platforms to self-align when they are brought in contact. In the previous iterations of this system, the mechanism consisted of a spring-loaded platform^3,33^. Although this approach enabled the two substrates to align at the beginning of the printing process, uncontrollable motion was observed during sliding (along the sliding direction) when using larger samples. Furthermore, relative rotation between the substrates along the axis perpendicular to the contact plane (z-axis) was observed (Supplementary Fig. S1). As a result, the uniformity of the electronic layer from the printed NWs was affected. In the presented system, the spring-loaded mechanism is replaced with a pair of purposefully designed platforms that are free to pivot about perpendicular axes, thus enabling planar alignment (Fig. 1b-v, vi). This self-aligning action of the substrate platforms when the force is applied is illustrated in Fig. 2b. Although the platforms are free to rotate about one axis, the design restricts any other motion. As a result, uniform contact is achieved across the whole printable area, the control of the sliding motion is significantly more robust, and the alignment of the printed NWs is not disturbed. An issue was identified when using small donor substrates, where the top platform would tilt out of alignment during sliding due to shear forces (Supplementary Fig. S2). To rectify this, an automated mechanism for fixing the top platform after the initial alignment was introduced (Fig. 1b-vii and Supplementary Movie 2). As such, the as-presented system allows a fully controlled printing process, and in the following sections, the study to assess the uniformity of printing is described along with the proposed printing setup.

### System operation for a highly controllable contact printing process

The contact printing system is operated through an in-house developed program created with the LabVIEW software development platform. The functions of the program are as follows: (a) to control each active component, (b) to execute the printing procedure in a closed-loop manner, and (c) to monitor each parameter throughout the printing process. As per the requirements of the system, the program allows control over various parameters of the printing process, namely the applied force within a certain error margin (0–6 N ± 0.3%), sliding velocity (0–200 mm/s), and sliding distance (0–100 mm). The applied force is the primary printing parameter and has an impact on printing performance, as discussed in a subsequent section. Although striving for uniform printing, it is essential to control and maintain the applied force at the prescribed level throughout the printing process. The presented system employs closed-loop control to maintain the prescribed force during the alignment, optional platform fixing, and printing/sliding procedures, as seen in Fig. 2c. The high accuracy of the load cell (4 × 10^−3^ kg) in conjunction with the high resolution (48 nm) of the vertical actuator allows for precise control over the applied force down to 0.02 N. A block diagram describing the closed-loop control implementation is shown in Fig. 2a. The sliding velocity and distance are the two parameters that are related to the throughput of the printing process. Specifically, sliding speed controls the time required to complete the process and sliding distance affects the resulting printed area. Controlling these parameters is important when considering a potential implementation of the contact printing process in an industrial setting. Additional features are incorporated into the control software to reduce errors and improve the repeatability of the printing procedure, including (a) automated calibration and (b) overload protection. These features in the presented system help reduce processing time, a key factor when carrying out large numbers of optimization tests, as is the intended use of the system.

## Discussion

Various studies conducted following the development of the described contact printing system are presented in this section. These studies aim to demonstrate the operation of the developed system, assess the printing process performance, and evaluate the design focusing on individual features. Through these studies, the system is used to gain a better understanding of the printing dynamics by examining the effects of various printing parameters. Specifically, studies were carried out to explore uniform printing across a large area (1 cm^2^), as well as the influence of applied pressure, sliding velocity, and receiver substrate material on the printing performance.

For the studies presented in this paper, ZnO NWs were used as the printed nanostructure material. ZnO NWs were synthesized using the previously reported bottom-up chemical vapor transport method^3,34^. SEM images of the donor substrates are shown in Supplementary Fig. S3. Following printing, SEM images of the printed samples were analyzed with open-source image analysis software^35^ to extract figures of merit (NW length, NW density, and NW alignment), which characterize the printing performance.

### Uniformity over a large area

The contact printing method holds great promise towards the large-scale fabrication of electronics. The system presented in this study was designed with this prospect in mind. The self-leveling substrate platforms are able to accommodate large samples and effectively align them for tight contact, which is critical for achieving large-area, uniform printing. To assess the printing uniformity and validate the design of the setup, 9 × 12 SEM images were taken across the entire printed NW-based electronic layer sample, covering an area of ~100 mm^2^. It should be noted that the presented sample is limited by the size of the donor and the system was designed to accommodate even larger samples.

**Fig. 3:**
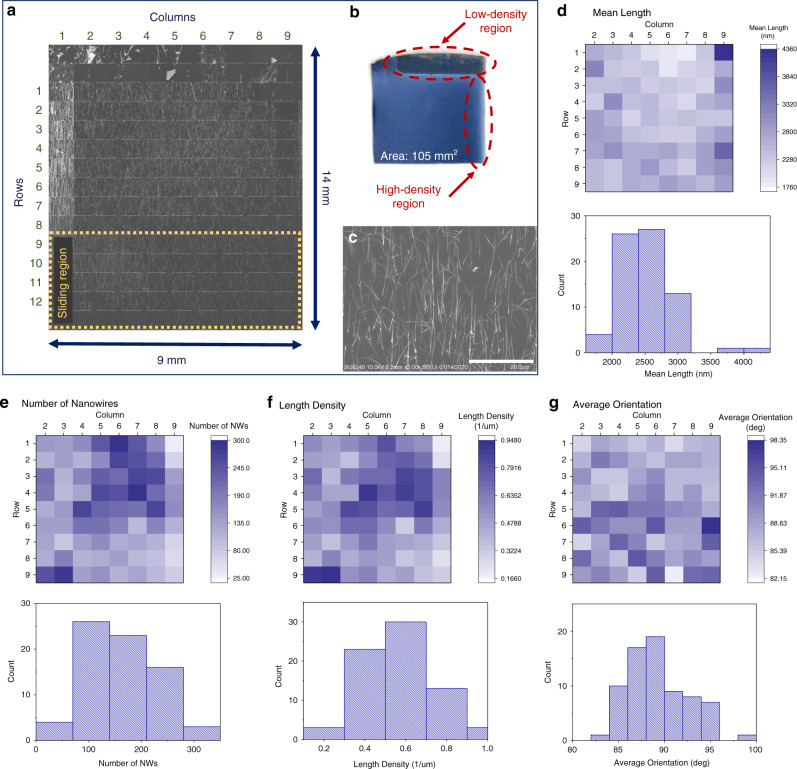
SEM study assessing the uniformity of the printed NWs. **a** Array of SEM images across the whole printed area of the receiver substrate. The sliding region represents the part of the receiver substrate that came in contact with the donor after sliding was initiated. **b** Image of the donor substrate showing regions of high and low density. Regions of high and low density can be observed at the corresponding locations on the receiver. **c** Higher magnification SEM image showing the printed nanowire layer and achieved alignment (scale bar 20 μm). **d** Mean nanowire length, **e** number of nanowires, **f** length unit density, and **g** average orientation. An orientation of 90° corresponds to nanowires oriented along the direction of sliding

For this study, a donor sample was used to print NWs on a Si receiver substrate. The printing pressure and sliding velocity were set to 33 kPa and 1 mm/s, respectively (see the influence of printing parameters in the following section). The sliding stroke is 5 mm. SEM images were acquired at 1 mm intervals over the whole printed area, as shown in Fig. 3a, whereas the donor sample is shown in Fig. 3b. By inspecting these images, it can be seen that the printed area extends across the entire area of the donor. This suggests that the system, via self-aligning platforms, is able to achieve tight contact between the donor and receiver substrates. Visual inspection also reveals regions of significantly low and high NW densities, which directly correspond to regions on the donor sample. The variations in the various figures of merit of the printed NWs, including the mean length, the length density, etc. are illustrated in Fig. 3d–g. Specifically, for more than 93% of the printed area, the average NW orientation is within ±5 deg of the sliding direction. This indicates a high level of NW alignment, which can also be seen from the SEM image in Fig. 3c. The SD for mean length equals ~15% of the mean value, whereas the equivalent figures for length density and NW count are 30% and 38%, respectively. These results indicate a good level of uniformity across the printed area for all figures of merit, except for the high-density region on the left-hand side, which is likely to originate from the non-uniformity in the donor substrate itself, not from the printing process. Nevertheless, this demonstrates that the system is able to achieve the printing of NWs with good uniformity by maintaining tight contact between the donor and receiver substrates. Further study will be focused on synthesizing a uniform large-area NW donor, which would potentially lead to a uniform large-area printing of NWs.

### Influence of the printing parameters

The contact printing process involves two main actuations: the pressing of the donor and receiver substrates and the relative sliding between the two. The presented system was designed to offer precise control over these two actuations and studies were carried out to independently assess the effect of such control over the applied pressure and sliding velocity.

**Fig. 4:**
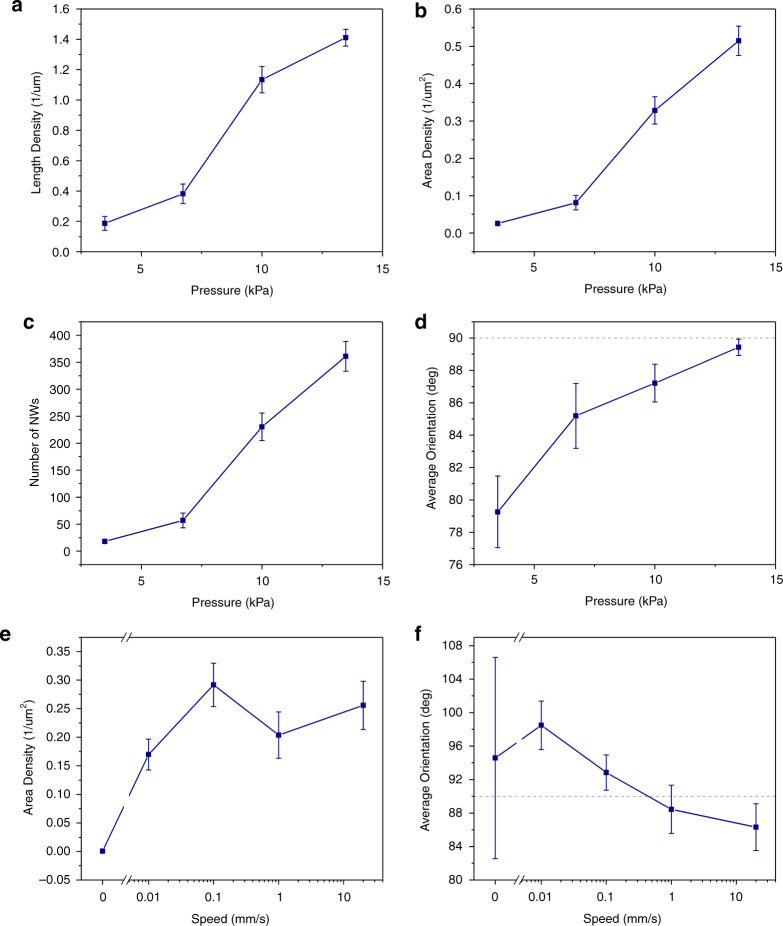
Influence of various printing parameters. Pressure control: **a** length unit density, **b** area density, **c** number of nanowires, and **d** average orientation. For the pressure control studies, the velocity was set to 1 mm/s. Velocity control: **e** area density and **f** average orientation. For the velocity control studies, the pressure was set to 10 kPa. The error bars correspond to standard error. An orientation of 90 deg corresponds to nanowires oriented along the sliding direction

To explore the influence of the printing pressure on the entire process, similarly sized donor samples were used (~400 mm^2^) and the experiments were performed across the entire range of the system, resulting in pressure values from 3.5 to 13.5 kPa. The sliding velocity was kept constant at 0.1 mm/s. Figure 4a–d illustrates the obtained results, from which it can be seen that the length density (Fig. 4a), area density (Fig. 4b), and number of NWs (Fig. 4c) increase significantly with increasing pressure. The average orientation is improved with increased pressure, whereas the orientation uniformity also improves, as indicated by the error bars (Fig. 4d). The results demonstrate the significance of the applied pressure during printing as a mechanism for breaking the NWs from the donor substrate and transferring them onto the receiver substrate. As previously shown through simulations^3^, NWs of different materials and dimensions require different applied forces to reach their breaking point. When considering a donor substrate with a slight variation in NW dimensions, there should exist a threshold of applied pressure above which the majority of NWs reach their breaking point and are hence transferred to the receiver substrate. The ability of the designed system to precisely control the applied pressure during printing allows for the optimal setting to be identified experimentally for different types of NWs. As a result, a high printing yield can be achieved while preventing negative effects that could result from excess pressure being applied, such as damage to the printed NWs or receiver substrates. Controlling the printing yield could also provide a means for tuning device performance. In addition, the ability of the system to maintain the prescribed pressure throughout the printing process minimizes the fluctuations in printing performance across the sample and therefore is an important factor for achieving uniform printing.

For the study on the sliding velocity, similarly sized donor samples were again used and sliding velocities greater than three orders of magnitude were tested. Specifically, the velocities ranged from 0.01 to 20 mm/s, whereas pressure was kept constant at ~10 kPa. The results are shown in Fig. 4e, f, where it can be seen that printing performance does not depend on sliding velocity, with both area density (Fig. 4e) and average orientation (Fig. 4f) remaining relatively unchanged. A sample where no sliding motion was used (velocity = 0) is also included in the results. This demonstrates the importance of the sliding motion and the resulting shear forces for the successful transfer and alignment of NWs. The sample without sliding motion demonstrates extremely low density and a large variation in alignment.

### Printing of NWs on flexible substrates

**Fig. 5:**
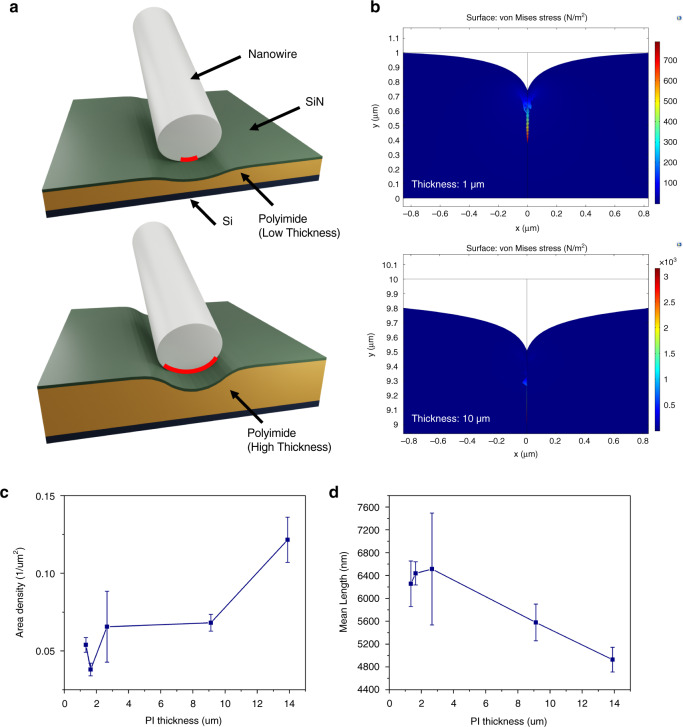
Nanowire printing on flexible substrates. **a** Schematic showing the change in contact area between the nanowire and substrate when changing the thickness of the polyimide layer. **b** Simulation results show the stress and deformation of the polyimide layers with different thicknesses when the same force is applied. Deformation is scaled for visual representation. The results for printing on flexible (polyimide) substrates of varying thickness: **c** length density and **d** mean length

When considering printed electronics, printing on flexible substrates is an important aspect, as many applications for these low-cost, high-volume devices would greatly benefit from such deformable form factors. The contact printing method, due to its simplicity and low temperature requirements, is compatible with flexible substrates. The developed system was designed to facilitate printing on different substrates. To demonstrate this capability, we printed NWs on polyimide (PI) substrates of varying thickness, ranging from 1.3 to 13.9 µm. An additional sample without PI was also included in the study. A layer of SiN (100 nm) was deposited on all receiver substrates prior to printing, to maintain identical surface conditions. Similar sized donor substrates were used and the applied pressure was kept the same (~9 kPa). Figure 5 shows the obtained results, where it can be seen that increasing the PI thickness increases the NW density (Fig. 5c), while the NW length is decreased (Fig. 5d), which may indicate that the receiver substrate could also influence the performance of the printed NWs. Specifically, a 100% increase in density is observed when the PI layer thickness increases from 9 to 14 μm, whereas the decrease in length is ~14%. This result can be qualitatively explained by the soft nature of the flexible PI layers with different thicknesses. For this, we developed a COMSOL model to simulate the printing process. The simulation results (Fig. 5b) for 1 and 10 µm-thick PI layers indicate that the vertical displacement is 35% smaller for the thinner layer when equal force is applied (see the Supplementary Information). As illustrated by the schematic in Fig. 5a, the increased deformation of the thicker PI layer can result in an increased contact area between the substrate and nanowires, thus increasing the transfer yield. Likewise, the surface morphology can also influence the printing of NWs, as evident from Supplementary Fig. S4. We realized three-dimensional features of different heights (but the materials were the same) and noted that when the feature height was sufficiently large, printing was achieved only at the regions between them. These results also show the potential of using surface morphology for printing NWs at desired or pre-defined locations^10^, which could be a promising direction to program NW printing on a location-to-location basis^9^. Nevertheless, further studies are needed to better understand this phenomenon. The printing performance for different NW materials and the influence of surface functionalization of the receiver substrate are also worth exploring. Some of the preliminary results demonstrate the successful printing of Si NWs (Supplementary Fig. S5), whereas O_2_ plasma treatment of the receiver substrate prior to printing results in a slight increase in printed NW density (Supplementary Fig. S6). This could be explained by the increase in the surface energy of the substrate due to the presence of volatile functional groups such as -OH and C = O, which form on the surface with O_2_ plasma treatment^36^.

### Arrays of printed ZnO NW-based UV photodetectors

**Fig. 6:**
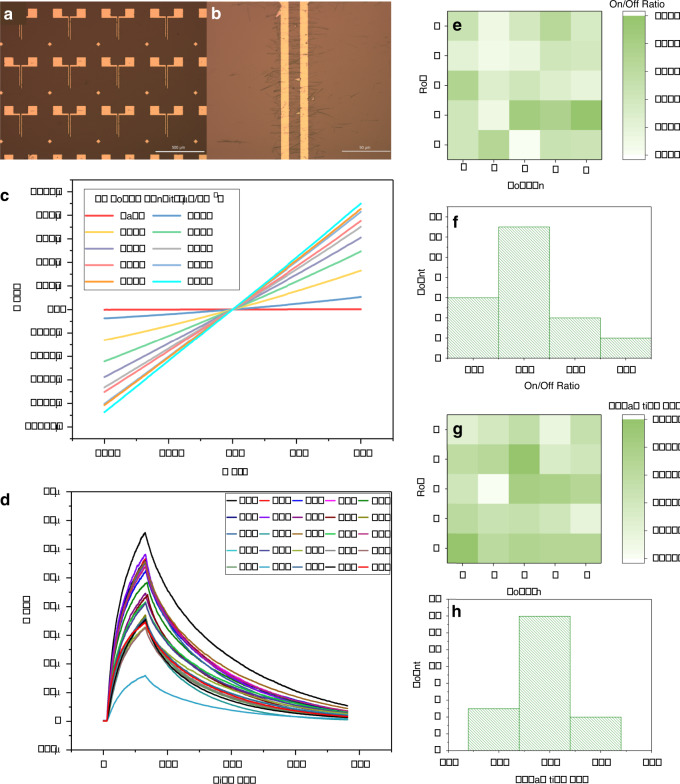
Characterization of printed nanowire-based devices. **a** Array of ZnO nanowire-based UV photodetectors fabricated with contact printing (scale bar is 500 μm). **b** Close-up image of the UV photodetector channel (scale bar of 50 μm). **c** *I*–*V* response of a single UV photodetector under different UV illumination power densities. **d** Single-cycle response of a 5 × 5 array of UV photodetectors. On/off ratio of a 5 × 5 array of UV photodetectors shown as a (**e**) spatial distribution and (**f**) histogram. Decay time of a 5 × 5 array of UV photodetectors shown as a (**g**) spatial distribution and (**h**) histogram

To further assess the printing uniformity achieved via the contact printing system, a 5 × 5 array of UV photodetectors based on a printed layer of ZnO NWs was fabricated (Fig. 6a). A close-up image of a single device reveals ~40 NWs bridging the 10 μm channel (Fig. 6b). The *I*–*V* response of a single device at different intensities of constant UV illumination is shown in Fig. 6c. The obtained response is linear, suggesting that the contact between the NWs and the electrodes is ohmic. Figure 6d illustrates the single-cycle response of all 25 devices measured at a UV current intensity of 0.01 A and voltage bias of 1 V. All devices show sensitivity to UV illumination at an average off current of ~150 nA and an average on current of 44 μA. The distributions of the on/off ratio and decay time across all devices are shown in Fig. 6e–h, respectively. The average decay time, defined as the time for the current to drop to 37% of the on current^21,37^, is ~193 s with an SD < 15% of the mean. The average on/off ratio is ~315 with an SD of ~28% of the mean. To the best of our knowledge, there has not been any study on the performance uniformity of such devices obtained with contact printing or other NW assembly techniques. A similar uniformity study was reported on NW-based UV photodetectors fabricated directly on a growth substrate using the step-corner growth mode^38^. When considering the on/off ratio uniformity, the obtained SD corresponds to ~55% of the mean, which indicates a larger variation compared to our contact printed devices. Overall, the obtained results indicate that the NW layers printed with the developed system have sufficient uniformity to successfully fabricate fully working arrays of printed devices with good performance uniformity.

## Conclusions

In summary, we presented the design and implementation of a custom-built system that implements the contact printing method for large-area printed electronics. The contact printing technique shows great potential to meet the requirements of the emerging printed electronics industry, offering a low thermal budget, large-scale compatibility, and reduced waste. The developed system aims to provide a highly controlled execution of the contact printing process (applied force: 0–6 N ± 0.3%, sliding velocity: 0–200 mm/s, and sliding distance: 0–100 mm) to achieve highly uniform large-area printing. A systematic study of the printing performance across an entire receiver substrate (1 cm^2^) revealed significant uniformity and validated the design choices. Further studies investigated the printing dynamics by exploring the effects of various printing parameters. NWs were aligned along 93% of the printed area, while the SD as a percentage of the mean for the average length, unit length density, and NW count were 15%, 30%, and 38%, respectively. The printing pressure presented a significant effect on the printing performance (sevenfold increase in NW density for a fourfold increase in pressure), while sliding velocity could be increased without causing major disturbances. The receiver substrate material also played a role and all parameters should be carefully selected and controlled to achieve the desired uniformity. The study of printing uniformity extended to the uniformity in device performance. The printed NW layers were used to fabricate a 5 × 5 array of fully working UV photodetectors with sufficient performance uniformity. The SD as a percentage of the mean for the decay time and on/off ratio was 15% and 28%, respectively. From developing the system for uniform printing and subsequently to device array fabrication, this work showcases the potential of contact printing for fabricating non-conventional electronics. Along with a better understanding of printing dynamics, it provides motivation for future works in redesigning the system based on a R2R approach, integrating it with other printing techniques and striving towards high-throughput printing of electronics.

## Materials and methods

### System components

For the vertical actuating platform, a Zaber (Canada) X-LSM050A motorized linear stage is used. The stage uses a two-phase stepper motor allowing for 0.047625 µm step movements and is capable of applying 25 N of continuous thrust. The controller of the stage is interfaced via the RS232 serial protocol. A custom-made machined aluminum bracket secures the linear stage in an upright position. The horizontal platform is actuated by a Motionlink (UK) motorized linear stage (IKO (Japan) TU25 positioning table, Maxon (Switzerland) brushless servo motor). The maximum achievable speed is 200 mm/s, and the loading capacity is 47 N. A Broadcom (USA) HEDL-5540 optical encoder enables a minimum step motion of 8 µm. The stage is operated by a Galil (USA) DMC-30012 controller interfaced via an RS232 serial protocol. A Tedea-Huntleigh (Israel) single-point load cell is used to monitor the force applied by the vertical platform. The load cell has a capacity of 0.6 kg and accuracy of 0.0067%. A Keysight (USA) E3631A bench power supply provides the excitation voltage, whereas a Keysight (USA) 34465A digital multimeter acquires the load cell measurement and transmits it via a USB connection. The screwing mechanism to prevent any misalignments of the two holding platforms during sliding is realized by a high-torque DC motor (Pololu (USA) 3057). The motor is operated by a bespoke Arduino-based controller and is interfaced via a UART serial protocol. A pair of digital microscopes (RS PRO (UK) USB Microscope) are used to monitor the alignment and printing process along both alignment axes in real time. The contact printing system is assembled on top of a Newport (USA) Vision IsoStation optical workstation with active isolation. All active components are connected to a master desktop computer system.

### Contact printing process

The printing process is carried out by means of the initial alignment and printing/sliding sub-procedures. During initial alignment, the donor and receiver substrates, mounted on the tilting platforms, are brought into contact. The prescribed force is applied, leading the free platforms to self-align. Tight contact is achieved between the two planar substrates, resulting in a maximum printable area, while making use of the entirety of the donor material. The stability of the force measurement is established before progressing to the next steps. Prior to the printing/sliding sub-procedure, an optional step of fixing the top platform is carried out. This ensures that tight contact between the two substrates is maintained throughout the printing process. The automation of the platform-fixing process prevents excess loading on the substrates. Subsequently, during the printing/sliding sub-procedure, the receiver substrate is displaced according to the prescribed distance and velocity. When completed, the donor and receiver substrates are separated by the lift-off technique. Significant data, in the form of force measurements and video, are recorded and displayed in real time during the complete process (see Supplementary Movie 1).

## Supplementary information

Supplementary - marked up

contact printing system in operation

mechanism for fixing the top substrate platform

## References

[1] Dahiya AS (2020). High-performance printed electronics based on inorganic semiconducting nano to chip scale structures. Nano Converg..

[2] Dahiya R (2019). Large-area soft e-skin: the challenges beyond sensor designs. Proc. IEEE.

[3] García Núñez C (2018). Heterogeneous integration of contact-printed semiconductor nanowires for high-performance devices on large areas. Microsyst. Nanoeng..

[4] Khan S, Lorenzelli L, Dahiya RS (2015). Technologies for printing sensors and electronics over large flexible substrates: a review. IEEE Sens. J..

[5] Wu W (2017). Inorganic nanomaterials for printed electronics: a review. Nanoscale.

[6] Carlson A, Bowen AM, Huang Y, Nuzzo RG, Rogers JA (2012). Transfer printing techniques for materials assembly and micro/nanodevice fabrication. Adv. Mater..

[7] García Núñez, C., Liu, F., Xu, S. & Dahiya, R. *Integration Techniques for Micro/Nanostructure-Based Large-Area Electronics*. *Elements in Flexible and Large-Area Electronics*, 10.1017/9781108691574 (Cambridge Univ. Press, 2018).

[8] Zumeit A, Navaraj WT, Shakthivel D, Dahiya R (2020). Nanoribbon‐based flexible high‐performance transistors fabricated at room temperature. Adv. Electron. Mater..

[9] Christou, A., Liu, F. & Dahiya, R. Assessing the stability of printed NWs by in situ SEM characterisation. In: *FLEPS 2020 - IEEE Int. Conf. Flex. Printable Sensors Syst*. 10.1109/FLEPS49123.2020.9239461 (2020).

[10] Roßkopf D, Strehle S (2016). Surface-controlled contact printing for nanowire device fabrication on a large scale. Nanotechnology.

[11] Fan Z (2008). Wafer-scale assembly of highly ordered semiconductor nanowire arrays by contact printing. Nano Lett..

[12] Yao J, Yan H, Lieber CM (2013). A nanoscale combing technique for the large-scale assembly of highly aligned nanowires. Nat. Nanotechnol..

[13] Yerushalmi R, Jacobson ZA, Ho JC, Fan Z, Javey A (2007). Large scale, highly ordered assembly of nanowire parallel arrays by differential roll printing. Appl. Phys. Lett..

[14] Fan Z (2009). Toward the development of printable nanowire electronics and sensors. Adv. Mater..

[15] Chang Y-K, Hong FC-N (2009). The fabrication of ZnO nanowire field-effect transistors by roll-transfer printing. Nanotechnology.

[16] Liu X, Long Y-Z, Liao L, Duan X, Fan Z (2012). Large-scale integration of semiconductor nanowires for high-performance flexible electronics. ACS Nano.

[17] Søndergaard RR, Hösel M, Krebs FC (2013). Roll-to-roll fabrication of large area functional organic materials. J. Polym. Sci. B Polym. Phys..

[18] Takahashi T (2009). Monolayer resist for patterned contact printing of aligned nanowire arrays. J. Am. Chem. Soc..

[19] Sun C (2010). Aligned tin oxide nanonets for high-performance transistors. J. Phys. Chem. C.

[20] Takei K (2010). Nanowire active-matrix circuitry for low-voltage macroscale artificial skin. Nat. Mater..

[21] Bai S (2011). High‐performance integrated ZnO nanowire UV sensors on rigid and flexible substrates. Adv. Funct. Mater..

[22] Wen L, Wong KM, Fang Y, Wu M, Lei Y (2011). Fabrication and characterization of well-aligned, high density ZnO nanowire arrays and their realizations in Schottky device applications using a two-step approach. J. Mater. Chem..

[23] Yu G (2013). Contact printing of horizontally-aligned p-type Zn3P2 nanowire arrays for rigid and flexible photodetectors. Nanotechnology.

[24] Liu H, Takagi D, Chiashi S, Homma Y (2010). Transfer and alignment of random single-walled carbon nanotube films by contact printing. ACS Nano.

[25] Chen G (2013). Single-crystalline p-Type Zn _3_ As_2_ nanowires for field-effect transistors and visible-light photodetectors on rigid and flexible substrates. Adv. Funct. Mater..

[26] Chen G (2014). High performance rigid and flexible visible-light photodetectors based on aligned X(In, Ga)P nanowire arrays. J. Mater. Chem. C.

[27] Javey A, Nam S, Friedman RS, Yan H, Lieber CM (2007). Layer-by-layer assembly of nanowires for three-dimensional, multifunctional electronics. Nano Lett..

[28] Fan Z, Ho JC, Jacobson ZA, Razavi H, Javey A (2008). Large-scale, heterogeneous integration of nanowire arrays for image sensor circuitry. Proc. Natl Acad. Sci. USA.

[29] Ford AC (2008). Synthesis, contact printing, and device characterization of Ni-catalyzed, crystalline InAs nanowires. Nano Res..

[30] Takahashi T (2010). Parallel array InAs nanowire transistors for mechanically bendable, ultrahigh frequency electronics. ACS Nano.

[31] Liu Z (2013). Contact printing of horizontally aligned Zn2GeO4 and In2Ge2O7 nanowire arrays for multi-channel field-effect transistors and their photoresponse performances. J. Mater. Chem. C.

[32] Yao J (2014). Nanowire nanocomputer as a finite-state machine. Proc. Natl Acad. Sci. USA.

[33] Núñez CG, Vilouras A, Navaraj WT, Liu F, Dahiya R (2018). ZnO nanowires-based flexible UV photodetector system for wearable dosimetry. IEEE Sens. J..

[34] Shakthivel, D., Ahmad, M., Alenezi, M. R., Dahiya, R. & Silva, S. R. P. *1D Semiconducting Nanostructures for Flexible and Large-Area Electronics: Growth Mechanisms and Suitability*, 10.1017/9781108642002 (Cambridge Univ. Press, 2019).

[35] Persson NE (2017). High-throughput image analysis of fibrillar materials: a case study on polymer nanofiber packing, alignment, and defects in organic field effect transistors. ACS Appl. Mater. Interfaces.

[36] Alam AU, Howlader MMR, Deen MJ (2014). The effects of oxygen plasma and humidity on surface roughness, water contact angle and hardness of silicon, silicon dioxide and glass. J. Micromech. Microeng..

[37] Law JBK, Thong JTL (2006). Simple fabrication of a ZnO nanowire photodetector with a fast photoresponse time. Appl. Phys. Lett..

[38] Gao Z (2018). Improving the fabrication uniformity of ZnO nanowire UV sensor by step-corner growth mode. Ceram. Int..

