# Correction: Development of a highly controlled system for large-area, directional printing of quasi-1D nanomaterials

**DOI:** 10.1038/s41378-021-00326-2

**Published:** 2021-11-26

**Authors:** Adamos Christou, Fengyuan Liu, Ravinder Dahiya

**Affiliations:** Bendable Electronics and Sensing Technologies (BEST) Group, James Watt School of Engineering, University of Glasgow, Glasgow, G12 8QQ UK

**Keywords:** Electrical and electronic engineering, Nanowires

Correction to: *Microsystems & Nanoengineering*

10.1038/s41378-021-00314-6 published online 19 October 2021

Following publication of this article^1^, it is reported the Acknowledgement section need to be updated as below:

This work is partly supported by the EPSRC through an Engineering Fellowship (EP/R029644/1) and Programme Grant-Heteroprint (EP/R03480X/1) and North West Centre for Advanced Manufacturing project funded by the European Union’s INTERREG programme (H2020-Intereg-IVA5055), managed by the Special EU Programmes Body. The views and opinions in this document do not necessarily reflect those of the European Commission or the SEUPB.

The original article has been updated.
