# Supplementary material for: Development of a highly controlled system for large-area, directional printing of quasi-1D nanomaterials
Source: Microsyst Nanoeng. 2021 Oct 19;7:82. doi: 10.1038/s41378-021-00314-6 (PMC8523549; doi:10.1038/s41378-021-00314-6)
Supplement: Supplementary file 1 — Supplementary - marked up [file 41378_2021_314_MOESM1_ESM.docx]

# **Development of a highly controlled system for large-area, directional printing of quasi-1D nanomaterials**

**Supplementary Information**

Adamos Christou, Fengyuan Liu and Ravinder Dahiya*

### Comparison of contact printing implementations

Table S1: Comparison of contact printing implementations with respect to the information on printing parameters, system automation, scanning electron microscopy (SEM) analysis and device uniformity.

| **Ref** | **Printing parameters** | | | **System automation** | **SEM analysis** | **Device uniformity** |
| --- | --- | --- | --- | --- | --- | --- |
|  | **Speed** | **Pressure** | **Stroke** |  |  |  |
| ^1^ | 6 mm/s | 15kPa | N/A | Motorized sliding actuator,  Manual pressure application (weight) | Single SEM | No information provided |
| ^2^ | 0.33 mm/s | 0.98 kPa  1.96 kPa | 0.5mm | Mechanism assisted sliding*,  Manual pressure application (weight) | Single SEM | No information provided |
| ^3^ | N/A | N/A | N/A | No information provided | Single SEM | No information provided |
| ^4^ | N/A | N/A | N/A | No information provided | No SEM | I_on_ Std Dev 13% of the mean (40 devices)** |
| ^5^ | N/A | N/A | N/A | No information provided | Single SEM | Photocurrent Std Dev 39% of the mean (208 devices)** |
| ^6^ | N/A | N/A | N/A | No information provided | Single SEM | No information provided |
| ^7^ | N/A | N/A | N/A | No information provided | No SEM | No information provided |
| ^8^ | 5 mm/s | 50 kPa | N/A | No information provided | Single SEM | No information provided |
| ^9^ | N/A | N/A | N/A | No information provided | No SEM | No information provided |
| ^10^ | 0.33 mm/s | 0.98 kPa  5.3 kPa | N/A | Mechanism assisted sliding*,  Manual pressure application (weight) | Single SEM | No information provided |
| ^11^ | N/A | N/A | N/A | No information provided | Single SEM | No information provided |
| ^12^ | 0.33 mm/s | 0.98 kPa  5.39 kPa | 1 mm | Mechanism assisted sliding*,  Manual pressure application (weight) | Single SEM | No information provided |
| ^13^ | N/A | N/A | N/A | No information provided | Single SEM | No information provided |
| ^14^ | 0.083 mm/s | 1.96 kPa | 1 cm | Mechanism assisted sliding*,  Manual pressure application (weight) | Single SEM | No information provided |
| ^15^ | N/A | N/A | N/A | No information provided | Single SEM | No information provided |
| ^16^ | N/A | 5 kPa | 1 mm | Manual pressure application (by hand) | Single SEM | No information provided |
| ^17^ | N/A | N/A | N/A | No information provided | Single SEM | No information provided |
| ^18^ | 0.083 mm/s | 19.6 kPa | N/A | Mechanism assisted printing* | Single SEM | No information provided |

*No information provided on automated operation

**Estimated data

### Uncontrollable motion in spring-loaded self-aligning mechanism during printing


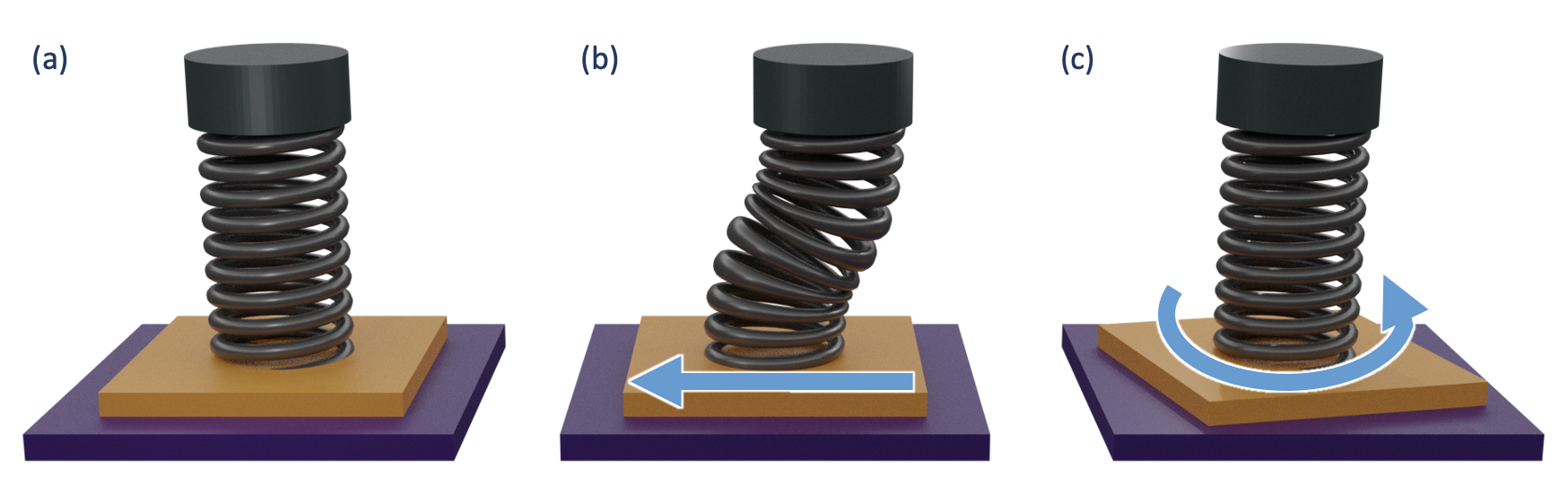


Fig. S1: Unwanted movement observed in spring-loaded self-aligning substrate platform. (a) Starting position. (b) Uncontrolled motion along the direction of sliding. (c) Uncontrolled rotation about the vertical axis.

### Top substrate holder platform tilting out of alignment during printing


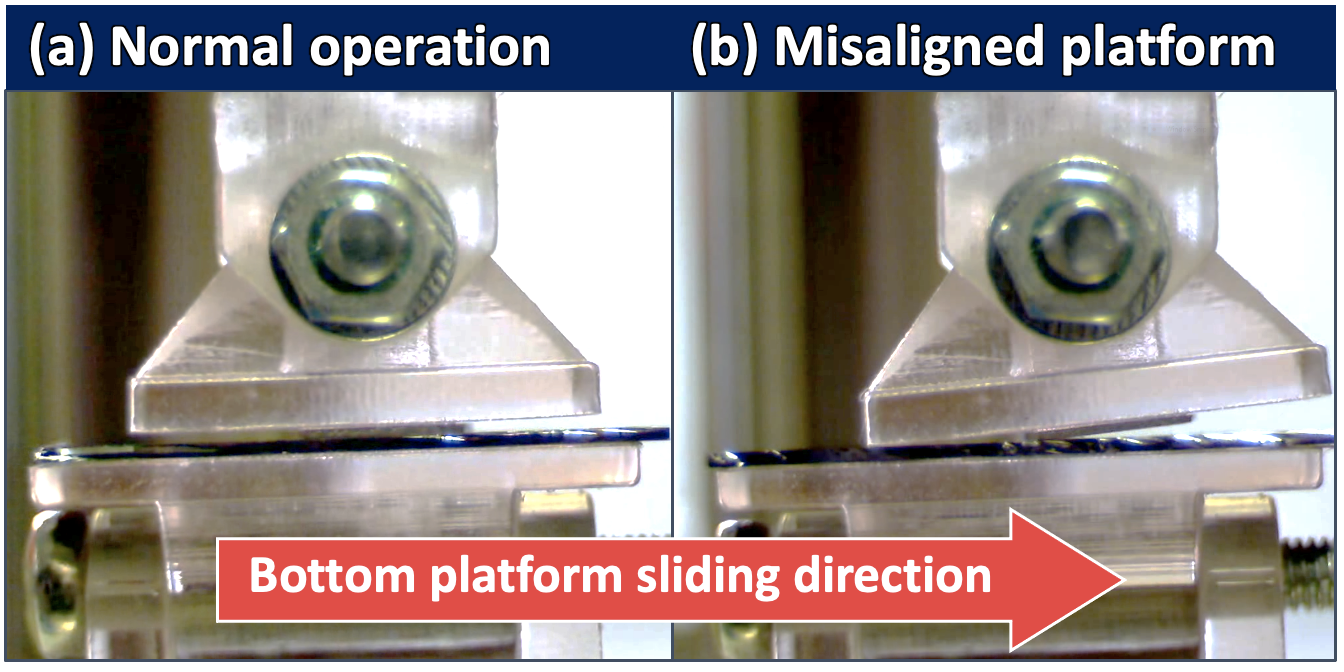


Figure S2: Self-aligning substrate platforms under (a) normal operation and (b) misalignment of the top platform due to the shear forces resulting from the sliding of the bottom platform.

### SEM images of ZnO nanowire donors

###
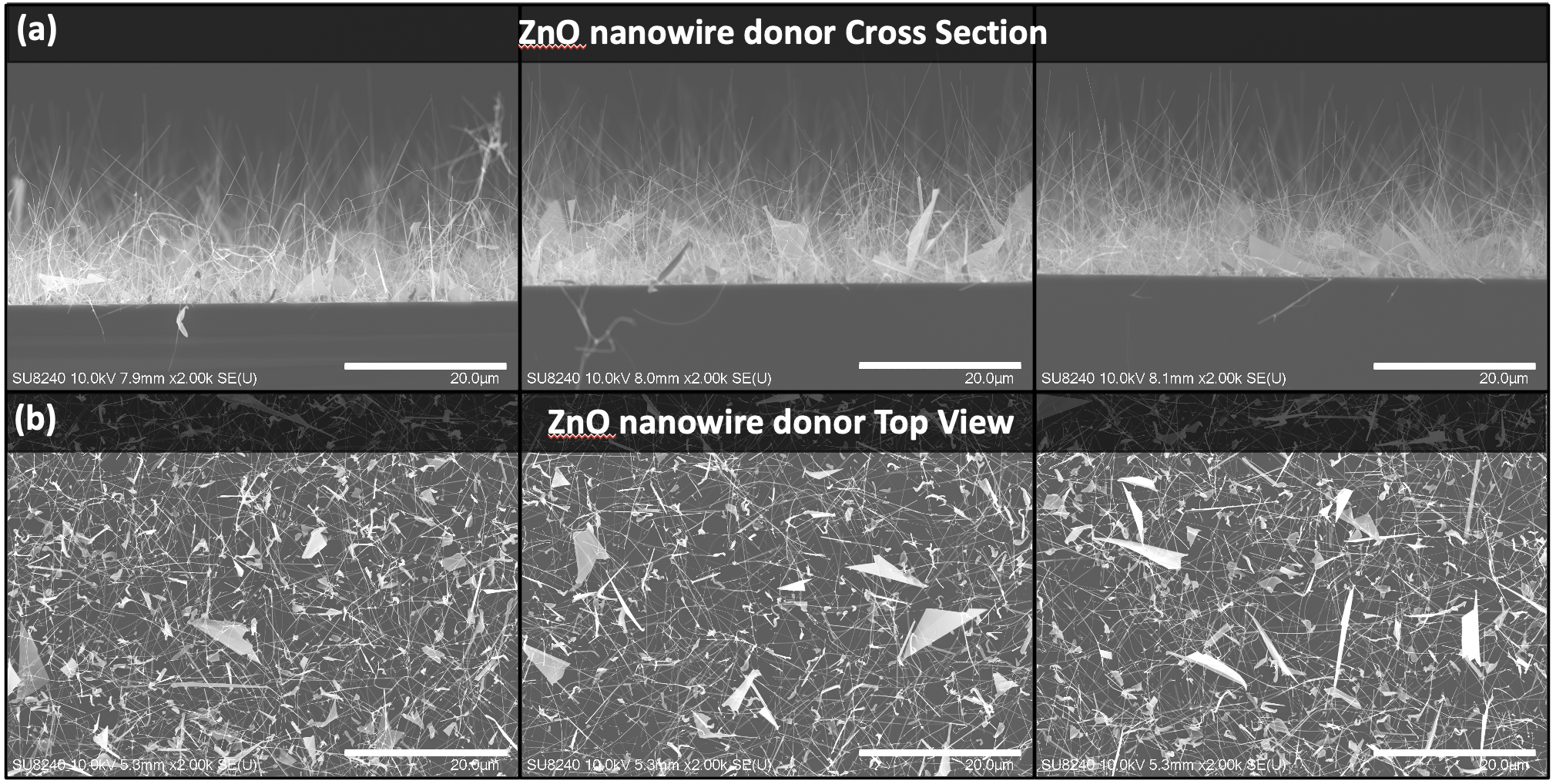


Figure S3: SEM images of ZnO nanowire donor taken at different locations. (a) Cross section, (b) Top view. Scale bar: 20um.

### Simulation of printing process on flexible substrates of varying thickness

The simulation was carried out using the COMSOL Multiphysics software. A 2D linear elastic model of the flexible polyimide (PI) layer was studied. The simulated model was 10um wide and two different thicknesses were tested, 1um and 10um, corresponding to the thickness of the PI layers obtained via spin-coating which were used during the printing experiments. A point load was applied at the center of the top surface corresponding to the 10 kPa pressure applied during the experiments. The maximum vertical displacement, located under the point load, was extracted from the simulation results and was found to be 35% smaller for the thinner PI layer.

### Influence of surface morphology on printing performance

A preliminary study was carried out to investigate how surface morphology of the receiver substrate affects the printing performance. To this end, some 3D features were created on receiver substrates by depositing patterned Au layers of different thicknesses. The surface was covered with 100 nm Si3N4 to exclude any impacts other than surface morphology (Fig.S4a). While using nanowires (NWs) of 100 nm diameter and feature height of 200nm, NWs are not effectively printed on top of the features (peaks) but tend to gather in the valleys (Fig.S4b). Lower density was observed at wider valleys (Fig.S4c) and higher density at narrower valleys (Fig.S4d). Further studies are required to investigate the influence of surface morphology on printing performance and its potential exploit for location-controlled printing.


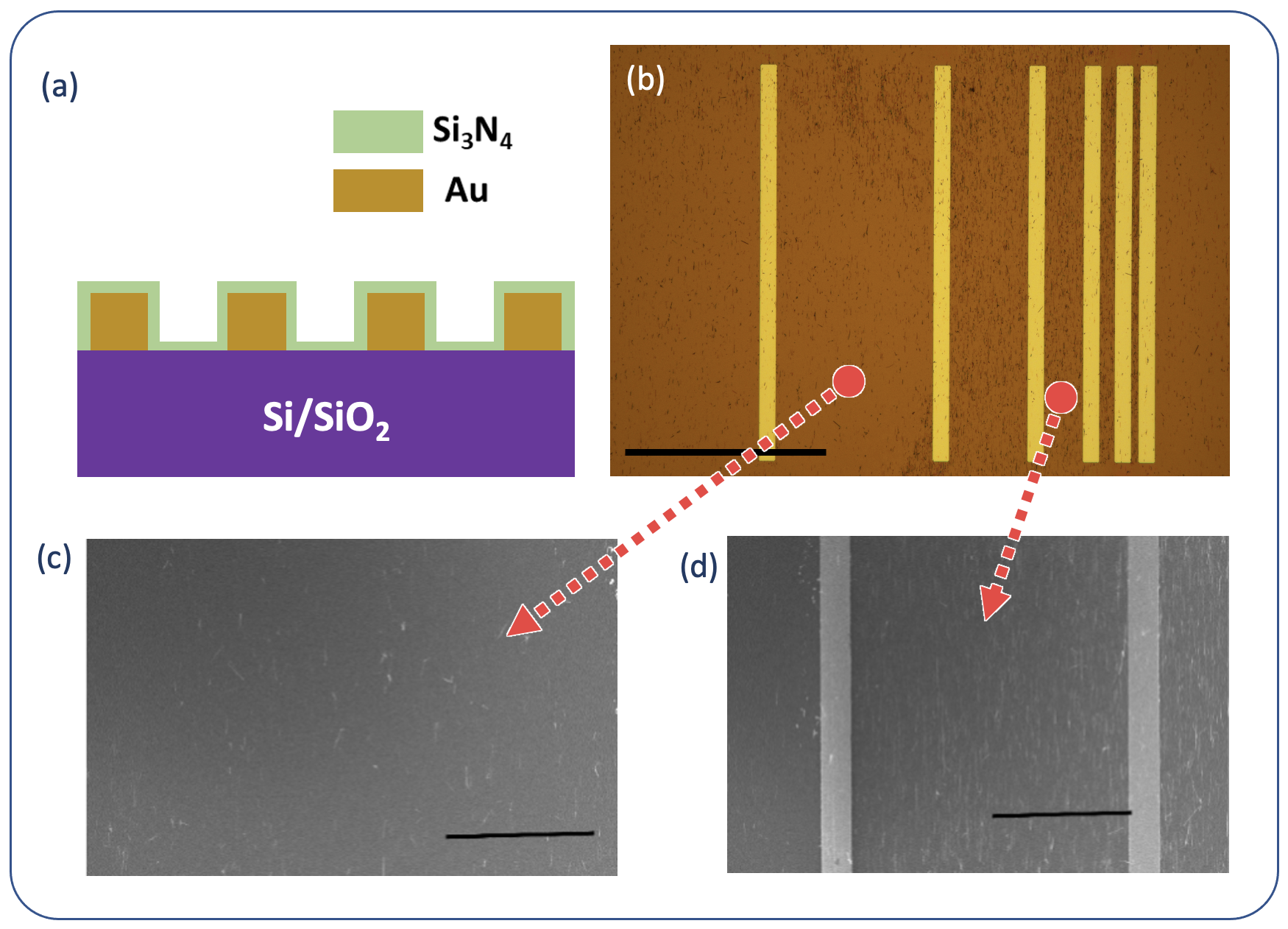


Fig. S4: (a) Diagram of receiver substrate with 3D features to study the influence of surface morphology. (b) Optical microscope image of nanowires (d=100nm) printed on uneven surface with feature height 200nm. Scale bar: 250μm. (c) SEM image of nanowires printed at wider region between 3D features (valleys). Scale bar: 50μm. (d) SEM image of nanowires printed at narrower region between 3D features showing higher density. Nanowires are not printed on top of the 3D features (peaks). Scale bar: 50μm.

### Printing of Si Nanowires


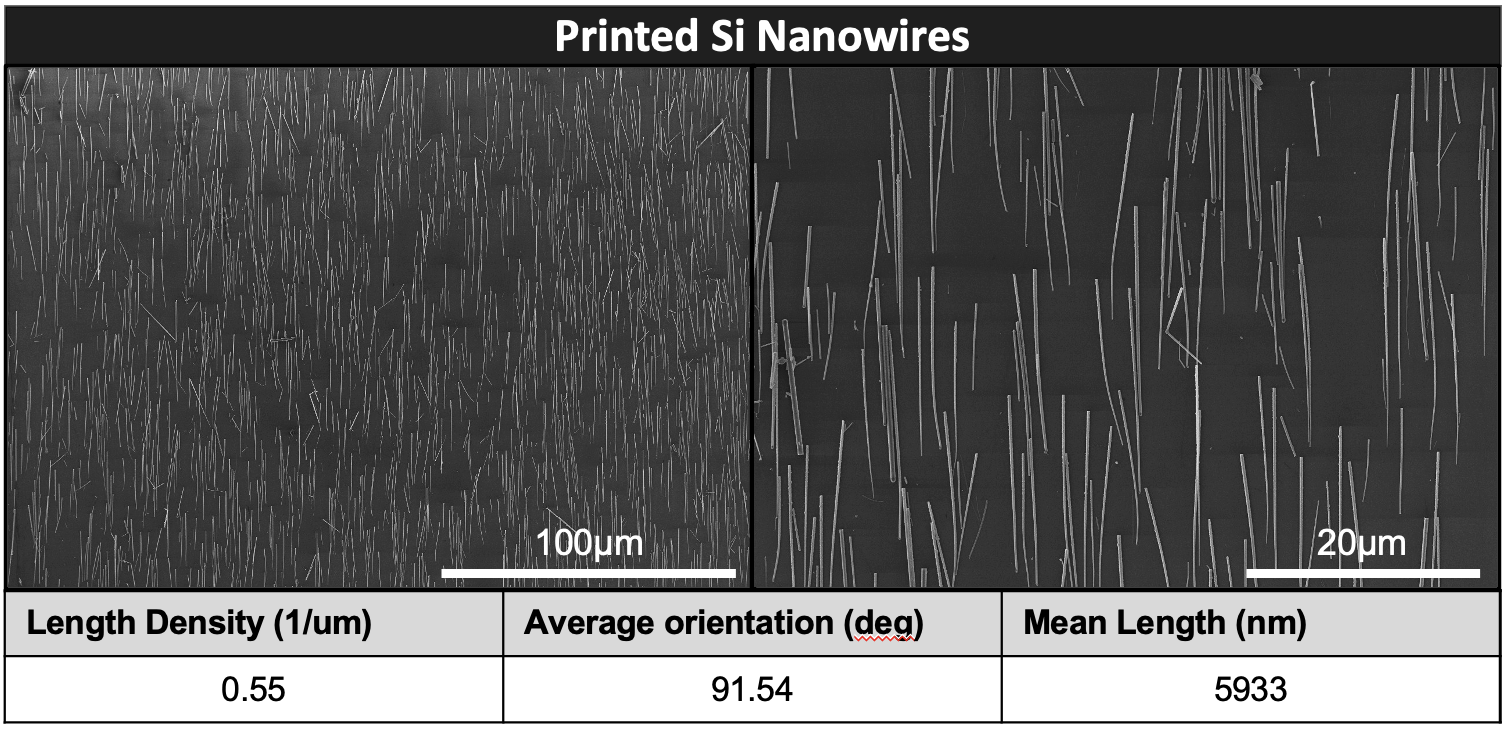


Figure S5: Contact printed Si nanowires

Si NWs were printed using the developed contact printing system. As seen in Fig.S5, the Si NWs were successfully transferred and aligned on the receiver substrate, thus demonstrating the compatibility of the system with other NW materials. The Si NWs were synthesized using a bottom-up chemical vapor transport (CVT) method. Since the diameter of the Si NWs was larger than the ZnO NWs used in the other studies and the tensile strength of Si is larger than ZnO, a higher pressure (83 kPa) was applied during printing^19^. Although printing was successful, the Si NW donor samples had poor uniformity compared to the ZnO NW donors, hence the latter were used for the characterization studies presented in this paper.

### Influence of O_2_ plasma treatment on the printing performance


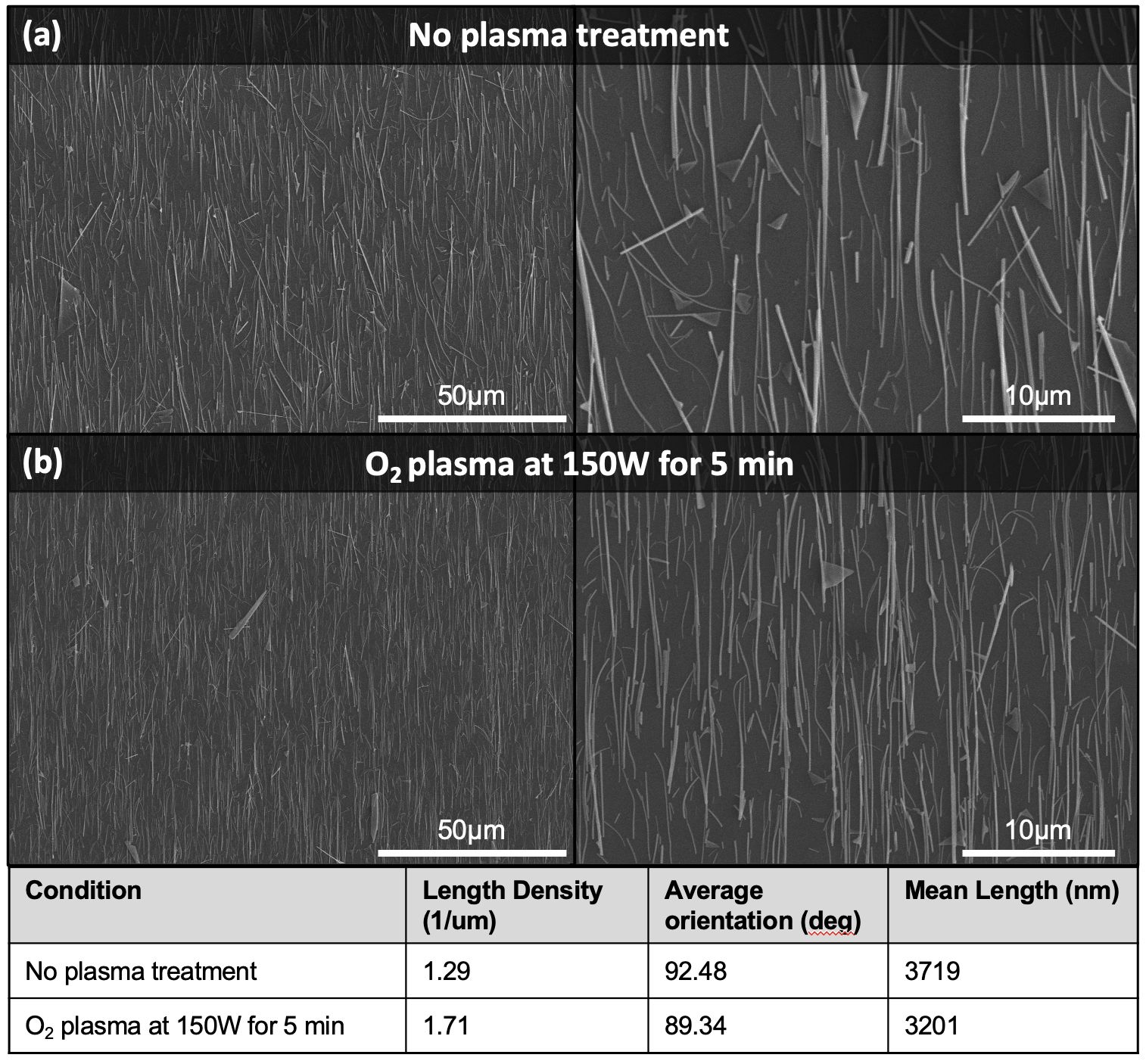


Figure S6: Influence of O_2_ plasma treatment on the printing performance. (a) Printed sample without plasma treatment. (b) Printed sample with O_2_ plasma treatment at 150W for 5 min.

This experiment represents a preliminary study on the influence of surface functionalization of the receiver substrate on the printing performance. A SiO_2_ receiver substrate was treated with O_2_ plasma at 150W for 5 min prior to printing. Fig.S6 compares the printing performance against a receiver substrate which did not undergo any treatment. A slight increase in NW density is observed on the sample with plasma treatment. This could be explained as with the oxygen plasma treatment, volatile functional groups such as -OH and C=O form on the surface and the surface energy of the substrate increases. It is worth noting that although plasma treatment could provide a means for increasing the printed NW density, the additional step can have an impact on the printing uniformity across the printed area. Further studies are required to better understand the phenomenon.

### Supplementary Movie 1

Video showing the developed contact printing system in operation. Close-up views of the self-aligning platforms along the two axes of alignment demonstrate the mechanism’s operation. The applied force measurement is also shown as obtained in real time.

### Supplementary Movie 1

Video showing the mechanism for fixing the top substrate platform in operation. When the top platform is not fixed, slight misalignment is observed while printing. The mechanism is used to fix the top platform after initial alignment to prevent misalignment during printing while also keeping the disturbances in the applied force to a minimum.

## References

1. Roßkopf, D. & Strehle, S. Surface-controlled contact printing for nanowire device fabrication on a large scale. *Nanotechnology* **27**, 185301 (2016).

2. Fan, Z. *et al.* Wafer-Scale Assembly of Highly Ordered Semiconductor Nanowire Arrays by Contact Printing. *Nano Lett.* **8**, 20–25 (2008).

3. Chen, G. *et al.* High performance rigid and flexible visible-light photodetectors based on aligned X(In, Ga)P nanowire arrays. *J. Mater. Chem. C* **2**, 1270–1277 (2014).

4. Javey, A., Nam, S., Friedman, R. S., Yan, H. & Lieber, C. M. Layer-by-Layer Assembly of Nanowires for Three-Dimensional, Multifunctional Electronics. *Nano Lett.* **7**, 773–777 (2007).

5. Fan, Z., Ho, J. C., Jacobson, Z. A., Razavi, H. & Javey, A. Large-scale, heterogeneous integration of nanowire arrays for image sensor circuitry. *Proc. Natl. Acad. Sci. U. S. A.* **105**, 11066–70 (2008).

6. Ford, A. C. *et al.* Synthesis, contact printing, and device characterization of Ni-catalyzed, crystalline InAs nanowires. *Nano Res.* **1**, 32–39 (2008).

7. Takahashi, T. *et al.* Parallel Array InAs Nanowire Transistors for Mechanically Bendable, Ultrahigh Frequency Electronics. *ACS Nano* **4**, 5855–5860 (2010).

8. Liu, Z. *et al.* Contact printing of horizontally aligned Zn2GeO4 and In2Ge2O7 nanowire arrays for multi-channel field-effect transistors and their photoresponse performances. *J. Mater. Chem. C* **1**, 131–137 (2013).

9. Yao, J. *et al.* Nanowire nanocomputer as a finite-state machine. *Proc. Natl. Acad. Sci.* **111**, 2431–2435 (2014).

10. Takahashi, T. *et al.* Monolayer Resist for Patterned Contact Printing of Aligned Nanowire Arrays. *J. Am. Chem. Soc.* **131**, 2102–2103 (2009).

11. Sun, C. *et al.* Aligned Tin Oxide Nanonets for High-Performance Transistors. *J. Phys. Chem. C* **114**, 1331–1336 (2010).

12. Takei, K. *et al.* Nanowire active-matrix circuitry for low-voltage macroscale artificial skin. *Nat. Mater.* **9**, 821–826 (2010).

13. Bai, S. *et al.* High‐Performance Integrated ZnO Nanowire UV Sensors on Rigid and Flexible Substrates. *Adv. Funct. Mater.* **21**, 4464–4469 (2011).

14. Wen, L., Wong, K. M., Fang, Y., Wu, M. & Lei, Y. Fabrication and characterization of well-aligned, high density ZnO nanowire arrays and their realizations in Schottky device applications using a two-step approach. *J. Mater. Chem.* **21**, 7090–7097 (2011).

15. Yu, G. *et al.* Contact printing of horizontally-aligned p-type Zn3P2 nanowire arrays for rigid and flexible photodetectors. *Nanotechnology* **24**, 95703 (2013).

16. Liu, H., Takagi, D., Chiashi, S. & Homma, Y. Transfer and Alignment of Random Single-Walled Carbon Nanotube Films by Contact Printing. *ACS Nano* **4**, 933–938 (2010).

17. Chen, G. *et al.* Single-Crystalline p-Type Zn _3_ As _2_ Nanowires for Field-Effect Transistors and Visible-Light Photodetectors on Rigid and Flexible Substrates. *Adv. Funct. Mater.* **23**, 2681–2690 (2013).

18. Yerushalmi, R., Jacobson, Z. A., Ho, J. C., Fan, Z. & Javey, A. Large scale, highly ordered assembly of nanowire parallel arrays by differential roll printing. *Appl. Phys. Lett.* **91**, 203104 (2007).

19. García Núñez, C. *et al.* Heterogeneous integration of contact-printed semiconductor nanowires for high-performance devices on large areas. *Microsystems Nanoeng.* **4**, 22 (2018).
